# Polyphenol-Rich Foods for Human Health and Disease

**DOI:** 10.3390/nu12020400

**Published:** 2020-02-03

**Authors:** María-Teresa García-Conesa, Mar Larrosa

**Affiliations:** 1 Quality, Safety and Bioactivity of Plant Foods, Food Science and Technology Department, CEBAS-CSIC, P.O. Box 360, Campus de Espinardo, Espinardo, 30100 Murcia, Spain; 2 Food, Microbiota and Health Group, Department of Pharmacy and Biotechnology, Faculty of Biosciences, Universidad Europea de Madrid, c/Tajo s/n Villaviciosa de Odón, 28670 Madrid, Spain; mar.larrosa@universidadeuropea.es

Polyphenols are a class of well-known bioactive compounds widely distributed in the plant kingdom and abundant in plant foods and derived food products. The biological activity of polyphenols have been long investigated using a variety of in vitro and in vivo experimental models that have attributed to these compounds a range of properties with the potential to contribute to maintain health and to prevent, delay, or reduce some chronic diseases (cardiovascular, inflammation, neurological disorders, etc.,) [1]. Nevertheless, and after several decades of research in this area, there are still many unresolved questions. The evidence of the benefits of the intake of polyphenols or polyphenol-rich foods in humans is still limited partly because of the large interindividual variability in response to the consumption of these compounds. We are only beginning to pin down some of the factors influencing this variability and, it is now clear that we still need to understand how these factors influence the response to polyphenols as well as to identify which individuals would benefit most from the intake of these compounds [2]. The mechanisms of action underlying the response to polyphenols intake are also rather complex since they appear to be multiple (via regulation of gene expression and (or) protein activity), occur at different body sites (gastrointestinal tract, various host organs), and the responsible molecules have not yet been fully identified [3]. Research in the area of polyphenols and human health should continue focusing on: (1) Proving the effects of polyphenols consumption in humans by confirming the associated beneficial regulatory changes in specific disease-associated biomarkers in well-defined target populations, and (2) elucidating the mechanisms of action and the responsible molecules triggering these mechanisms (original plant compounds, derived metabolites). This Special Issue of *Nutrients* contributes toward these goals by gathering a total of 13 articles trying to give response to some of these topics.

The identification of those individuals that could benefit most from the intake of polyphenols is not a trivial matter. This Special Issue includes two stimulating clinical trials looking at the effects of some of these compounds in two specific human sample subpopulations, i.e., individuals at cardiovascular risk [4] and postmenopausal women [5]. Accumulated evidence of the modulatory effects of polyphenol-containing berries on cardiometabolic health suggests that these compounds may reduce total-cholesterol (T-Chol) and (or) blood pressure (BP) [6]. The study by Pokimica et al. [4] was elegantly designed to specifically investigate the effects of a chokeberry juice with two (high- and low-) doses of polyphenols (mostly anthocyanins) against a nutritionally matched polyphenol-free placebo in individuals well characterized by having some cardiovascular risk. The results show a lack of effect of the daily intake for several weeks of the chokeberry juice on a range of classic anthropometric and cardiovascular biomarkers such as T-Chol and BP [4]. The second trial [5] also indicates the absence of effect on various inflammatory and metabolic biomarkers, including BP, following the consumption for several months of anthocyanins in post-menopausal women. Notwithstanding all the limitations and differences between the two studies, these results show the difficulty in demonstrating consistent responses of the cardiovascular biomarkers to intervention with dietary polyphenols. An insufficient number of participants, small effect sizes, and residual high variability (likely caused by other potential factors) in the specific subpopulations investigated may be some of the reasons behind this lack of clear detectable effects but, it is also plausible that these specific groups of individuals do not truly benefit from the consumption of this type of polyphenols. Indeed, an increasing number of intervention studies are now reporting the lack of effects of specific polyphenols in different individuals and disorders [7,8]. Understanding interindividual variability remains essential to fully comprehend the potential benefits of polyphenols against the development of chronic diseases in humans. It should also be noted that the biomolecules truly responding to the intake of these compounds may not have been yet fully identified and that as suggested by Pokimica et al. [4], the fatty acid (FA) composition (proportion of saturated (SFA) and polyunsaturated (PUFA)) may be a suitable responsive biomarker, and thus, the metabolism of FA in different tissues in response to polyphenols should be further and more closely investigated. 

Along these lines, the article published by García-Contreras et al. [9] focuses on the effects of maternal supplementation with hydroxytyrosol on the metabolism of lipids in fetuses of Iberian sows and shows that it modifies the PUFA profile of the fetal liver and muscle. That the hepatic lipid metabolism may be involved in the response to polyphenols is further supported by Rafiei et al. [10] that use a well-known in vitro model of liver (HepG2 cells) to screen for the ability of different polyphenols to reduce oleic acid-induced steatosis by modulating the expression of several key genes involved in lipid and FA metabolism. Further and complex mechanistic studies are also included in this Special Issue where Cuyàs et al. [11] propose a new mechanism of action for oleacein, a phenolic compound present in extra virgin olive oil. These authors combine computational, enzyme activity, and cell experimental models to show oleacein modulates the activity of a histone demethylase (an epigenetic regulator involved in different chronic human diseases) and of the expression of genes controlled by this enzyme. A common feature of these two late studies is that they use relatively low µM concentrations of the test polyphenols representing an effort toward more physiologically relevant experimental approaches. A further step into this direction is given by Pourová et al. [12] who investigated and compared the vasorelaxant and antiplatelet effects of sylimarin flavonolignans and of their sulfate conjugates. The study indicates that a particular metabolite, silychristin-19-*O*-sulfate, displays the best vasodilatory activity and reinforces the relevance of deciphering the bioactivity of the polyphenol-derived metabolites. With regards to the multiplicity and difficulty of establishing the mechanisms of action of polyphenols, the review presented by Rothenberg and Zhang [13] very smartly shows the biological complexity underlying a pathological process such as depression and, how different polyphenols present in tea can influence a diversity of pathways associated with this disease contributing this way to moderate the process. This review includes an interesting section dedicated to the gut-brain axis, the influence of microbiota composition on the brain chemistry and how tea polyphenols can have a large impact of this relationship as a mechanism to modulate mental health and depression. 

This Special Issue also includes a couple of excellent articles in relation with the exciting research area of the binomial polyphenols-gut microbiota (GM). Of particular interest is the article published by Gomes et al. [14] in which using a rat model of salt-induced hypertension, the authors demonstrate that the cardiovascular events promoted by the high salt consumption are ameliorated by the intake of mix berries rich in polyphenols. Very importantly, the authors find that the increase of BP promoted by the salt intake is associated with changes in the GM and in some specific short-chain FA that are reverted by the consumption of the berries suggesting that interaction between berry compounds and (or) their metabolites and the GM function and metabolism might be involved in the regulatory effects of the polyphenol-rich berries. These authors also provide a meticulous description of all the berry-derived metabolites detected in the urine and fecal samples which becomes extremely useful for future research to try to identify which molecule(s) might be responsible for the observed effects. In addition to this, the Issue presents an updated and very clear review of the current knowledge about GM and its role in health and disease as well as about how dietary polyphenols may modify the composition and functionality of the GM and how this may be related to the beneficial properties of these compounds [15]. The authors complete the review by also indicating the need to develop new and better strategies to improve the delivery of polyphenols to their target sites and cells thus improving their efficacy within the intestine or other host inner tissues. In this manner, the article published by Gracia et al. [16] shows how impregnation of the polyphenol curcumin into a biodegradable polymer increases the anticancer activity of this compound in a xenograft animal model of prostate cancer. Deciphering the metabolic fate of polyphenols and enhancing their bioavailability to target tissues will contribute greatly to increase our understanding of their beneficial effects against human diseases.

The search for the potential mechanisms of action of polyphenols goes on and thus, this Special Issue also includes two more exploratory pre-clinical studies, one looking at the effects of a grape seed extract rich in proanthocyanidins in colon permeability and its repercussion on visceral pain [17] and, a second one looking at the effects of the consumption of a high-molecular-weight polyphenol-rich fraction from black tea on muscle mass recovery after induced atrophy [18]. In both studies the authors also try to associate the observed phenotypic responses with modulation of specific key molecular markers of inflammation [17] and of central regulatory pathways of cell growth and metabolism such as mTOR (mechanistic target of rapamycin kinase), respectively [18]. This late pathway is considered a clinical target of great interest for the treatment of different chronic pathologies such as cancer, inflammatory processes, or diabetes [18].

Last, but not least, this Issue includes a stimulating review proposing new hypotheses in relation with the consumption and metabolism of polyphenols and their health benefits. The idea is to incorporate the concept of chrononutrition, i.e., the study of the interactions between biological rhythms, metabolism, and nutrition, into the research area of polyphenols and health [19]. It seems reasonable that biological rhythms which are present in all organisms (plant and animals) and include both circadian and seasonal rhythms may influence the human responses to the intake of dietary polyphenols. This research should combine the bidirectional understanding of the influence of biological rhythms on the plant production and composition in polyphenols as well as on the metabolic and responsive capacity of the consumers. This way, chrononutrition becomes another interesting factor that contributes to human interindividual variability in response to polyphenols and that surely needs further and extensive investigation.

The selection of articles included in this Special Issue show some of the current progress on the knowledge about the effects of plant dietary polyphenols on human health as well as the complexity of some of the issues that remain to be understood. It also highlights the need for further human clinical trials with better designs to understand interindividual variability and to improve the consistency and relevance of the effects in humans. It additionally shows the difficulty but, also the importance, of understanding the metabolism and mechanisms of action of these compounds and the interest in translating this knowledge into improved technologies to enhance the efficiency of the application of polyphenols for human health and disease.
